# Comparison of HER2-Targeted Antibodies for Fluorescence-Guided Surgery in Breast Cancer

**DOI:** 10.1155/2021/5540569

**Published:** 2021-02-02

**Authors:** Solmaz AghaAmiri, Jo Simien, Alastair M. Thompson, Julie Voss, Sukhen C. Ghosh, Servando Hernandez Vargas, Sarah Kim, Ali Azhdarinia, Hop S. Tran Cao

**Affiliations:** ^1^The Brown Foundation Institute of Molecular Medicine, McGovern Medical School, The University of Texas Health Science Center at Houston, TX 77054, USA; ^2^Division of Molecular and Clinical Medicine, Ninewells Hospital and School of Medicine, University of Dundee, Dundee DD1 9SY, UK; ^3^Michael E. DeBakey Department of Surgery, Baylor College of Medicine, Houston, Texas 77030, USA; ^4^Department of Surgical Oncology, The University of Texas M.D. Anderson Cancer Center, Houston, Texas 77030, USA

## Abstract

**Background:**

Although therapeutic advances have led to enhanced survival in patients with human epidermal growth factor receptor 2 (HER2)-positive breast cancer, detection of residual disease remains challenging. Here, we examine two approved anti-HER2 monoclonal antibodies (mAbs), trastuzumab and pertuzumab, as potential candidates for the development of immunoconjugates for fluorescence-guided surgery (FGS).

**Methods:**

mAbs were conjugated to the near-infrared fluorescent (NIRF) dye, IRDye800, and for quantitative *in vitro* assessment, to the radiometal chelator, desferrioxamine, to enable dual labeling with ^89^Zr. *In vitro* binding was evaluated in HER2-overexpressing (BT474, SKBR3) and HER2-negative (MCF7) cell lines. BT474 and MCF7 xenografts were used for *in vivo* and *ex vivo* fluorescence imaging.

**Results:**

*In vitro* findings demonstrated HER2-mediated binding for both fluorescent immunoconjugates and were in agreement with radioligand assays using dual-labeled immunoconjugates. *In vivo* and *ex vivo* studies showed preferential accumulation of the fluorescently-labeled mAbs in tumors and similar tumor-to-background ratios. *In vivo* HER2 specificity was confirmed by immunohistochemical staining of resected tumors and normal tissues.

**Conclusions:**

We showed for the first time that fluorescent trastuzumab and pertuzumab immunoconjugates have similar NIRF imaging performance and demonstrated the possibility of performing HER2-targeted FGS with agents that possess distinct epitope specificity.

## 1. Introduction

Breast cancer is the most common cancer and second leading cause of cancer deaths among women in the United States, with an estimated incidence of 268,600 new cases and estimated deaths surpassing 41,760 in 2019 [1]. A central component of breast cancer treatment is complete surgical extirpation of the tumor, along with adjuvant therapies that may include radiation therapy, chemotherapy, endocrine therapy, and targeted therapy. It has been well established that when surgical resection is incomplete and surgical margins are positive, the risk for ipsilateral breast tumor recurrence is more than doubled [2]. Since invasive disease and ductal carcinoma in situ (DCIS) lesions are often impalpable, surgeons often not only rely on preoperative imaging for tumor localization but also require assistance with three-dimensional spatial orientation in the operating room to determine which and how much tissue to excise. Difficulties in intraoperative tumor visualization may lead to insufficient resection with positive margin(s) or, conversely, excessive removal of tissue that can be disfiguring.

Fluorescence-guided surgery (FGS) is an emerging imaging technology with the potential to improve surgical, oncologic, and oncoplastic outcomes by augmenting the ability of surgeons to delineate cancer from noncancerous tissue in real-time and enabling intraoperative detection of tumors that are invisible to the naked eye. Near-infrared fluorescence (NIRF) imaging is particularly advantageous for FGS because of the low tissue autofluorescence in this range (750-900 nm) and the subsequent ability to obtain higher tumor-to-background ratios (TBRs). Additionally, NIRF imaging offers better depth penetration and can detect lesions as deep as 2-4 cm beneath the tissue surface [2]. Indocyanine green (ICG) is the only FDA-approved NIRF dye and has been used intraoperatively to recognize critical structures such as the ureters [3] and the bile duct [4] and the assessment of vascular perfusion of flaps [5] and bowel segments [6]. Since ICG is not suitable for bioconjugation, considerable efforts in NIRF dye development have produced new reagents that can be readily combined with biomolecules for tumor-specific imaging. Peptides and monoclonal antibodies (mAbs) have served as the primary classes of targeting agents for FGS probe development, with FDA-approved mAbs providing an expedited pathway toward clinical studies based on their known targeting properties and safety profiles. Notable examples include FGS with cetuximab (antiepidermal growth factor receptor) in head and neck cancers, pancreatic cancer, and malignant glioma [7–9] and with bevacizumab (antivascular endothelial growth factor) for pancreatic adenocarcinoma, breast cancer, and several other types of tumors [10, 11].

Human epidermal growth factor receptor 2 (HER2) is a tyrosine kinase-dependent receptor whose amplified expression is associated with poor prognosis in breast cancer. HER2 is overexpressed in 20-25% of breast cancers [12] and leads to tumors that are clinically aggressive. Compared with HER2-negative breast cancers, HER2-positive breast cancers have historically had a high rate of recurrence and distant metastasis that result in a poor prognosis. HER2 is an important biomarker as it allows patients to undergo anti-HER2 therapy with clinically approved mAbs, particularly trastuzumab and pertuzumab, for both localized and disseminated diseases. HER2 also plays an important role in noninvasive imaging as shown by the application of radiolabeled analogs of trastuzumab and pertuzumab for staging, treatment monitoring, and assessing HER2 status on primary and metastatic lesions by positron emission tomography (PET) [13, 14]. Anti-HER2 mAbs have also been fluorescently labeled with a variety of dyes to assess their potential for FGS; however, a direct comparison between the two therapeutic antibodies using the most clinical relevant NIRF dye for bioconjugate development, IRDye800, is lacking. The goal of this study was to evaluate the *in vitro* and *in vivo* characteristics of IRDye800-labeled trastuzumab and pertuzumab in breast cancer cell lines and xenografts with varying HER2 expression levels as a prelude to clinical studies.

## 2. Materials and Methods

### 2.1. Reagents and General Methods

All reagents were of analytical grade and used without further purification unless otherwise stated. Chelex-100 resin was purchased from Bio-Rad Laboratories (Richmond, CA) and used with aqueous buffers for radiolabeling experiments to ensure metal-free conditions. Trastuzumab and pertuzumab were obtained from Genentech (San Francisco, CA). Control IgG1 antibodies, 88R20 and mAb-69 were kindly provided by Dr. Kendra Carmon and Dr. Barret R. Harvey, the University of Texas Health Science Center at Houston. IRDye800CW-NHS was purchased from LI-COR Biosciences (Lincoln, NE). Desferrioxamine-*p*-benzyl-isothiocyanate (DFO-Bz-NCS) was purchased from Macrocyclics (Plano, TX). Zirconium-89 (^89^Zr)-oxalate was produced by Washington University School of Medicine (St. Louis, MO). Size-exclusion high-performance liquid chromatography (SEC-HPLC) was performed on an analytical Hitachi LaChrom system using a TSKgel G3000SW (5 *μ*m) column and mobile phases of A = 0.1 M sodium phosphate buffer (pH 7.3) and B = CH_3_CN (isocratic: 90% A and 10% B) and a flow rate of 1 mL/min. Radiochemical yield and purity were measured with a radio-thin-layer chromatography (TLC)/HPLC detector system (LabLogic) using previously described methods [15].

### 2.2. Synthesis of IRDye800-Labeled mAbs

Trastuzumab and pertuzumab were conjugated with IRDye800CW-NHS ester according to published procedures [16]. Briefly, a four-fold molar excess of IRDye800-NHS in dimethylsulfoxide (DMSO) (5 mg/mL) was added to a solution of pertuzumab, trastuzumab, or a control mAb in PBS (2 mg/mL) with 0.1 M Na_2_CO_3_ buffer (pH 9). The reaction was stirred at room temperature for 2 h and continued at 4°C overnight. The product was separated from unreacted dye with a 7 kDa Molecular weight cut-off (MWCO) Zeba desalting column and collected in PBS. Concentrations were determined using a NanoDrop spectrophotometer (Thermo Scientific), and purity was assessed by HPLC.

### 2.3. Synthesis of Dual-Labeled mAbs

IRDye800-mAb conjugates were dual-labeled with ^89^Zr for quantitative radioactive uptake studies according to previously described methods [17]. Briefly, 1 mg of each fluorescent immunoconjugate was reacted with an 8-fold molar excess of DFO-Bz-NCS in 0.1 M sodium carbonate (pH 9.0) buffer. Reactions were performed at 37°C for 1 h with continuous stirring and purified with Zeba desalting spin columns as described above. ^89^Zr-oxalate was diluted with an equal volume of 0.5 M HEPES buffer. The pH of the radioactive solution was adjusted to 7.4 with 2 N NaOH, and 37 MBq of the ^89^Zr-oxalate was added to 100 *μ*g of each immunoconjugate in 1 M HEPES buffer. After heating at 37°C for 1 h, the reaction was quenched with 10 mM ethylenediaminetetraacetic acid (EDTA) and purified with Zeba desalting spin columns. Radiochemical yield and purity were measured by instant-TLC and HPLC, respectively. To determine the ratio of the IRDye-800 to the mAb and the number of DFO molecules per immunoconjugate, mass spectrometry was performed (Agilent 6538 Ultra high definition Accurate-mass Q-Tof).

### 2.4. Cell Lines and Animal Models

The human breast cancer cell lines SKBR3, BT474, and MCF7, were obtained from the American Type Culture Collection (ATCC). Cells were cultured in Dulbecco's Modified Eagle's Medium (DMEM) with 10% (*v*/*v*) fetal bovine serum (FBS) and incubated at 37°C with 95% humidity and 5% CO_2_ atmosphere. Athymic female nu/nu mice (Charles River Laboratories), aged 6-8 weeks, were housed in accordance with the Institutional Animal Care and Use Committee (IACUC) guidelines of The University of Texas Health Science Center at Houston and maintained on normal rodent diet. For xenografting, all mice were anesthetized with 1-2% isoflurane and then injected with 5 × 10^6^ cells resuspended in 100 *μ*L matrigel : PBS (1 : 1) on the left shoulder. Studies were performed when the tumor size reached an approximately 5-10 mm maximum diameter. Overdose of anesthesia followed by cervical dislocation was the method of euthanasia for mice in the terminal studies.

### 2.5. Saturation and Binding Affinity of Immunoconjugates

The equilibrium dissociation constant (*K*_d_) and half maximal inhibitory concentration (IC_50_) of the immunoconjugates were assessed by direct saturation binding and competition assays. For the saturation study, BT474 cells (HER2-positive) were seeded at the density of 1.5 × 10^5^ cells/well in 96-well plates and incubated with increasing concentrations (1.6 to 200 nM) of ^89^Zr-mAb or ^89^Zr-mAb-IRDye800 at 37°C for 90 min. To measure nonspecific binding, radio-immunoconjugates were coincubated with 4 *μ*M of the naked trastuzumab or pertuzumab in additional wells. In the competition study, mAb or mAb-IRDye800 (final concentration: 5 × 10^−6^ to 5 × 10^−2^ mg/mL) were mixed with either 12.5 nM of ^89^Zr-Trastuzumab or ^89^Zr-Pertuzumab, added to BT474 cells (1.5 × 10^5^ cells/well in 96-well plates) and incubated at 37°C for 90 min. In both studies, after the incubation time, cells were washed and resuspended in PBS and the total radioactivity was measured in a Wizard^2^ automated gamma counter (Perkin Elmer).

### 2.6. Flow Cytometry Analysis in Breast Cancer Cell Lines

Binding properties of IRDye800-immunoconjugates were studied in BT474 (HER2-positive) and MCF7 (HER2-negative) cells using flow cytometry on a NIRF-equipped BD FACSAria II. Cells were grown to 90% confluency and harvested in cell dissociation buffer. After washing, a total of 5 × 10^5^ cells were resuspended in 100 *μ*L of the growth media containing 10 *μ*g/mL of the corresponding immunoconjugate for 30 min at 4°C. Cells were then pelleted and washed three times with 200 *μ*L cold FACS buffer (PBS, 2% FBS. 0.1% NAN3), fixed in 4% paraformaldehyde, washed with PBS, and resuspended in 200 *μ*L PBS. The identical procedure was used with the isotype control IgG1, and untreated cells were used to evaluate background fluorescence. For blocking studies, cells were preincubated with 10-fold excess of the corresponding unconjugated antibody for 30 min at 4°C, pelleted, washed with FACS buffer, and underwent the same process as described above.

### 2.7. Radioactive Uptake Studies

Quantitative measurements of immunoconjugate uptake were performed using radioactive uptake studies as previously described [15]. In brief, BT474, SKBR3, and MCF7 cells were seeded into 96-well plates (200,000 cells/well) and incubated with 10 nM of trastuzumab and pertuzumab immunoconjugates possessing both IRDye800 and ^89^Zr labels at 37°C for 1 h. To evaluate specificity, blocking experiments were conducted by coincubating the dual-labeled agents with a 100-fold excess of the corresponding parent (unmodified) mAbs. Cells were then washed and collected, and radioactivity was quantified in a Wizard^2^ automated gamma counter (Perkin Elmer) to determine uptake as a percent of the total radioactivity added. Dual-labeled-IgG1 was used as a control to further demonstrate HER2 specificity. The total incubated radioactivity was determined from a known aliquot.

### 2.8. Confocal Microscopy

To further evaluate the binding and subsequent internalization of the immunoconjugates, immunofluorescent staining and confocal microscopy was performed. For immunofluorescent staining, BT474 cells were seeded at 1 × 10^5^ cells in 8-well culture slides (Falcon) and allowed to attach overnight. The next day, the media was removed, and cells were washed with PBS and incubated in 200 *μ*L media containing 20 *μ*g/mL of the mAb-IRDye800 conjugates and incubated at 4°C or 37°C for 2 h. At the end of the incubation, cells were washed twice with ice-cold PBS and fixed in 4% paraformaldehyde for 10 min at room temperature, washed twice, and mounted with Vectashield containing DAPI (Vector laboratories). Finally, the emitted fluorescent was visualized using a confocal microscope (Olympus FV3000). IRDye800 was detected using a 730 nm laser, and DAPI was detected using a 405 nm laser with appropriate filter settings. For blocking studies, BT474 cells were preincubated with 10-fold excess of the unconjugated trastuzumab or pertuzumab for 2 h at 4°C or 37°C, washed with PBS; then immunofluorescent study was performed similarly.

### 2.9. NIRF Imaging

The tumor targeting properties and biodistribution of the IRDye800-immunoconjugates (mono-labeled) were studied in BT474 and MCF7 xenografts. Animals (*n* = 3-5/group) were injected intravenously with 40 *μ*g of the trastuzumab-IRDye800, pertuzumab-IRDye800, or nonspecific IgG-IRDye800, and NIRF imaging was performed 48 h postinjection using the In-Vivo Xtreme (Bruker) optical imaging system. Excitation and acquisition parameters were the same throughout *in vivo* and *ex vivo* studies: excitation (760 nm), emission (830 nm), exposure time (1 s), FOV (19 cm), and f-stop (0.1). At the completion of *in vivo* imaging, mice were euthanized and selected organs were harvested and underwent *ex vivo* NIRF imaging. Region of interest (ROI) analysis was performed using the vendor software package (Molecular Imaging) to obtain tumor-to-tissue ratios (TBRs).

### 2.10. Tissue Processing and Immunohistochemistry (IHC)

Tissues from the *ex vivo* imaging studies were fixed in 10% natural buffered formalin for 24 h at room temperature and used to prepare formalin-fixed paraffin embedded (FFPE) blocks. FFPE blocks were then serially sectioned at 5 *μ*m thickness and dried overnight. Sections were deparaffinized with xylene and rehydrated in decreasing concentrations of ethanol, and one section per block was stained with H&E. To identify the HER2 expression, heat-induced antigen retrieval was performed on the selected slides using citrate buffer (pH 6.1), followed by IHC staining using a rabbit-specific HRP/DAB (ABC) detection kit (Abcam). Endogenous peroxidases were blocked for 30 minutes; after washing with PBS, slides were placed in a humid chamber and incubated with primary antibody (BioGenex) overnight at 4°C. Slides were rinsed in PBS and incubated with secondary antibody (biotinylated goat anti rabbit-polyvalent IgG) for 10 min at room temperature, then washed and treated with streptavidin peroxidase for 10 min. Tissue staining was visualized with a diaminobenzidine tetrahydrochloride (DAB) solution for 5 min. After washing in PBS, sections were counterstained with Mayer's hematoxylin (Fisher Healthcare), dehydrated through two changes of 96% and 100% alcohol, cleared in xylene, and cover-slipped with Cytoseal 60 mounting medium (Thermo Scientific). Sections were also imaged on an Odyssey slide scanner (LI-COR) to investigate the dye accumulation pattern within the tissues at 800 nm and the highest resolution [18].

### 2.11. Statistical Analysis

All experiments were performed in triplicates and results are presented as the mean ± standard deviation (SD). Group comparisons in radioactive uptake studies were performed with one-way analysis of variance (ANOVA) followed by the Holm-Sidak test for multiple comparisons. For ex vivo tissue fluorescence studies, group comparisons were analyzed using two-way ANOVA followed by the Holm-Sidak test. Statistical analysis was performed using GraphPad Prism version 8.0.2, and *P* value < 0.05 was considered statistically significant in all the experiments.

## 3. Results

### 3.1. Immunoconjugate Synthesis and Dual Labeling

IRDye800 was conjugated to the mAbs to produce fluorescent immunoconjugates with >90% purity as shown by the HPLC chromatograms in Sup. Fig. 1. Trastumzumab-IRDye800 and pertuzumab-IRDye800 had identical HPLC profiles, showing a major UV peak at 5.8 min and the corresponding fluorescence peak at 6.5 min. A small shoulder was observed on the UV traces but not on the fluorescence channel, suggesting minor aggregation of unlabeled mAb. For the dual-labeled mAbs, ^89^Zr radiolabeling was achieved with radiochemical yields of >80% and radiochemical purity > 99% (Sup. Fig. 1). Mass spectrometry results revealed the conjugation of the mAbs to DFO and IRDye800, resulting in approximately 2 DFO molecules per antibody. The dye : protein ratio was 2 and 1 for trastuzumb-IRDye800 and pertuzumab-IRDye800, respectively.

### 3.2. Saturation Binding and Affinity to HER2

The results of the direct (saturation) radioligand assay showed that the *K*_d_ values for ^89^Zr-trastuzumab and ^89^Zr-trastuzumab-IRDye800 were 18.25 and 26.4 nM, respectively. For ^89^Zr-pertuzumab and ^89^Zr-pertuzumab-IRDye800, *K*_d_ values were 27.69 and 43.55 nM, respectively. These results are similar to those shown in competition studies (Sup. Fig. 2 and 3) and collectively show that conjugation of IRDye800 to trastuzumab and pertuzumab does not significantly alter the binding affinity and specificity of the immunoconjugates.

### 3.3. Flow Cytometry Analysis of HER2 Binding

**Figure 1:**
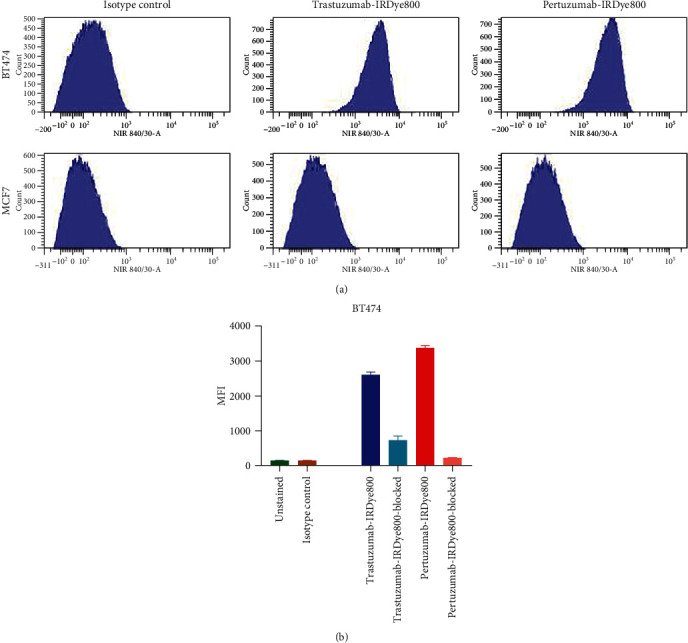
Flow cytometry analysis of IRDye800-immunocojugate binding in HER2-postive (BT474) and HER2-negative (MCF7) cells. HER2-specific binding was observed for trastuzumab-IRDye800 and pertuzumab-IRDye800 as indicated by a shift of the fluorescence signal to the right in BT474 cells compared to the lower fluorescence values measured from MCF7 cells (a). Plot of the MFI values of BT474 cells incubated with the immuoconjugates and the blocking doses of the unconjugated parental antibodies (b).

Flow cytometry was performed to examine antigen specificity of the mAbs after conjugation to IRDye800. Trastuzumab-IRDye800 and pertuzumab-IRDye800 specifically bound to HER2-positive cell lines as shown by the increased fluorescent intensity values compared with the isotype control (Figure 1(a)). As expected, BT474 cells with high (3+) expression of HER2 showed higher and similar binding of trastuzumab-IRDye800 and pertuzumab-IRDye800 compared to MCF7 cells with negative to low (0-1+) HER2 density. Blocking studies showed a distinct reduction in MFI values for the cells preincubated with the naked antibodies compared to the cells incubated with the immunoconjugates only (Figure 1(b)). The analysis of the fluorescent intensity showed that the binding of the trastuzumab-IRDye800 and pertuzumab-IRDye800 was inhibited about 71% and 93%, respectively, by preincubation with the unconjugated parental antibodies. This reduction shows the immunoconjugates bind specifically to the HER2 antigen and also they recognize the same epitope as their corresponding unconjugated antibodies.

### 3.4. Radioactive Uptake Studies

**Figure 2:**
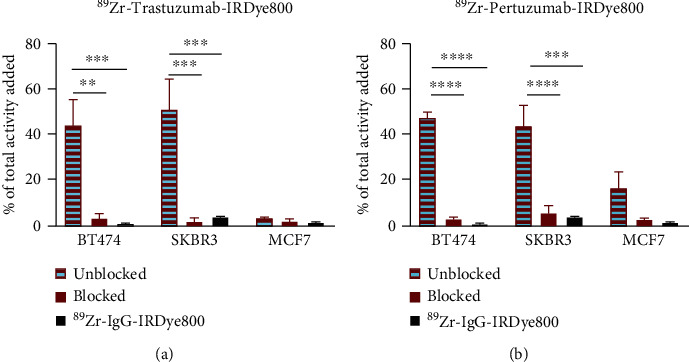
Quantitative assessment of HER2 binding with dual-labeled anti-HER2 immunoconjugates. Radioactive uptake of dual-labeled trastuzumab (a) and pertuzumab (b) correlated with HER2 expression and was blocked with a 100-fold excess of the corresponding naked mAb. Data are presented as the mean ± SD. ^∗∗^*P* < 0.01, ^∗∗∗^*P* < 0.001, and ^∗∗∗∗^*P* < 0.0001.

As shown in Figure 2, uptake of ^89^Zr-DFO-traztuzumab-IRDye800 and ^89^Zr-DFO-pertuzumab-IRDye800 was similar in each cell line and in agreement with HER2 expression levels. In BT474 cells, 44.6% ± 10.8 and 47.75% ± 2.05 of added ^89^Zr-DFO-traztuzumab-IRDye800 and ^89^Zr-DFO-pertuzumab-IRDye800 were taken up, respectively. Similar uptake was observed in SKBR3 cells (51.7% ± 12.58 and 44.05% ± 8.69, respectively), with notably lower accumulation in MCF7 cells (3.95% ± 0.07 and 16.75% ± 7.01, respectively). No significant uptake of the dual-labeled isotype control mAb was observed in the cell lines under the same conditions. Blocking studies with 100-fold unmodified mAb reduced binding of the dual-labeled HER2-targeted tracers by 93% and 94% in BT474 cells and by 97% and 88% in SKBR3 cells for ^89^Zr-DFO-traztuzumab-IRDye800 and ^89^Zr-DFO-pertuzumab-IRDye800, respectively. This reduction in the cellular uptake indicates that the dual-labeled mAbs maintain the antigen recognition characteristics of unconjugated trastuzumab and pertuzumab after bioconjugation.

### 3.5. Confocal Microscopy

**Figure 3:**
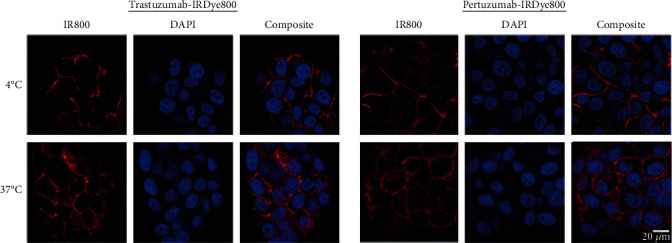
Confocal microscopy examination of cell surface binding and internalization of mAb-IRDye800 on BT474 cells at 4˚C (top) and 37˚C (bottom).

To show that IRDye800-conjugated trastuzumab and pertuzumab retain their functional activity to promote HER2 internalization after binding, confocal microscopy was performed on the immunostained BT474 cells at 4°C versus 37°C. A strong membrane staining was observed at 4°C, while internalization occurred at 37°C (Figure 3), indicating successful binding of the immunoconjugates to the cell surface HER2 and subsequent internalization of the bound immunoconjugates by BT474 cells. Competition of the immunoconjugates with the 10-fold excess of their unconjugated parental antibodies inhibited binding and internalization of the mAb-IR800Dye conjugates as shown in Sup. Fig. 4.

### 3.6. HER2-Targeted *In Vivo* Optical Imaging and Uptake Analysis

**Figure 4:**
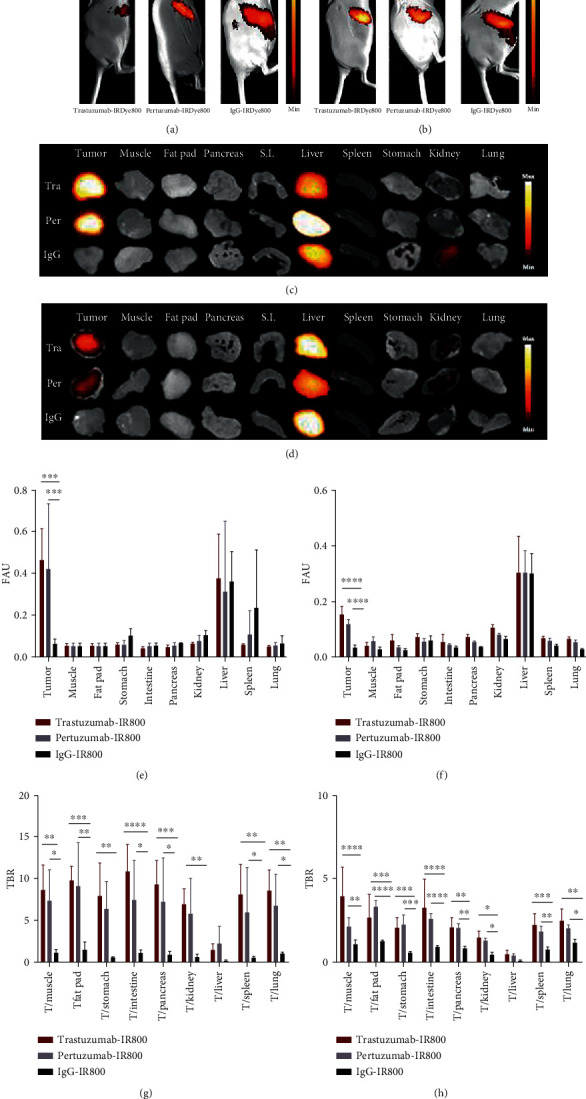
*In vivo* imaging of fluorescently-labeled trastuzumab and pertuzumab in mouse xenografts. Representative NIRF images at 48 h p.i. in BT474 (a) and MCF7 (b) xenografts show higher uptake in HER2-overexpressing tumors. *Ex vivo* images from corresponding BT474 (c) and MCF7 (d) mice confirm tumor uptake and show minimal signal in normal tissues. Quantification of tissue fluorescence and TBRs for BT474 (e, g) and MCF7 (f, h) mice. Data are presented as the mean ± SD. ^∗^*P* < 0.05, ^∗∗^*P* < 0.01, ^∗∗∗^*P* < 0.001, and ^∗∗∗∗^*P* < 0.0001. FAU: fluorescent arbitrary units; TBR: tumor to background ratio. Arrow indicates the tumor.

*In vivo* NIRF imaging with IRDye800-conjugated trastuzumab and pertuzumab demonstrated clear tumor uptake of the immunoconjugates in HER2-overexpressing BT474 xenografts, whereas minimal signal was detected in MCF7 tumors (Figures 4(a) and 4(b)). No tumor-associated fluorescence was observed in mice injected with the control mAb. Fluorescence signal was also evident in the abdomen due to hepatic uptake and elimination of the mAbs. *Ex vivo* images were consistent with the *in vivo* findings and showed that no significant fluorescence signal was present in nontarget tissues (Figures 4(c) and 4(d)). The analysis of the *ex vivo* images revealed equal fluorescence intensities for trastuzumab-IRDye800 and pertuzumab-IRDye800 in BT474 tumors, which were significantly higher than the control mAb (*P* < 0.05) (Figure 4(e)). As expected, MCF7 xenografts showed lower tumor accumulation compared to BT474 xenografts for both HER2-targeted immunoconjugates (3.06-fold for trastuzumab-IRDye800 and 3.81-fold for pertuzumab-IRDye800) Figure 4(f). In normal tissues that are clinically relevant sites for HER2-targeted FGS, low background uptake of immunoconjugates resulted in TBRs of 10.10 ± 1.50 and 9.31 ± 5.09 (mammary fat pad), 8.70 ± 2.30 and 7.37 ± 3.69 (muscle), and 8.00 ± 3.90 and 6.43 ± 3.27 (stomach), for trastuzumab-IRDye800 and pertuzumab-IRDye800, respectively, in BT474 mice (Figure 4(g)). Even in well-perfused organs, such as lung, TBRs of 8.7 ± 2.3 and 6.9 ± 3.8 were achieved for trastuzumab-IRDye800 and pertuzumab-IRDye800, respectively, indicating potential utility for intraoperative detection of metastases. Tumor uptake of HER2-targeted mAbs in MCF7 mice was likely due to the enhanced permeability and retention (EPR) effect and produced contrast ratios that were >2 in some organs (e.g., muscle, fat pad, small intestine, and lung) but were considerably lower than values obtained for BT474 mice (Figure 4(h)).

### 3.7. Histological and Fluorescent Confirmation of Tumor and Intratumoral Localization of Bioconjugates

**Figure 5:**
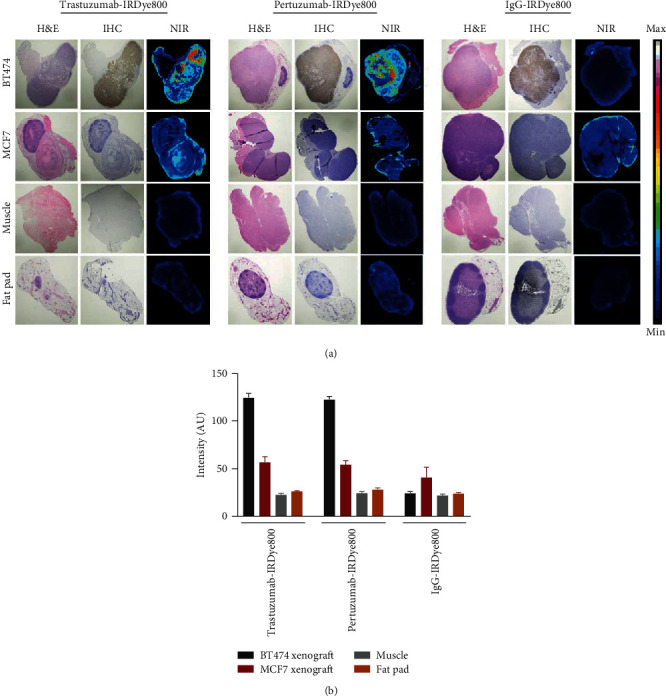
Histological analysis of immunoconjugate uptake. NIR fluorescence from IRDye800-labeled trastuzumab and pertuzumab correlated with IHC (a). Quantitative analysis of the fluorescence signal was higher in BT474 tumors compared to MCF7 tumors and normal tissues (b).

FFPE sections of tumors resected from mice injected with IRDye800 labeled mAbs were used to evaluate the relationship between localized NIRF signal and HER2 expression as shown in Figure 5. Consistent with *in vivo* findings, higher localized uptake of the HER2-targeted mAbs was observed in BT474 tumors compared to MCF7 tumors. Tissue staining showed that signal from the NIRF images was confined to areas with histologic evidence of tumor and demonstrated excellent colocalization with anti-HER2 IHC in BT474 tumors. Examination of probe uptake in muscle and fat pad sections revealed background-level fluorescence, suggesting low nonspecific binding for trastuzumab-IRDye800 and pertuzumab-IRDye800. Sections from mice injected with nonspecific isotype control showed low fluorescence in tumor and nontumor tissues. For trastuzumab-IRDye800 and pertuzumab-IRDye800, quantitative analysis of the tumor sections showed that fluorescence has 2.2-fold higher signal in BT474 tumors compared to MCF7 tumors (Figure 5(b)) and resulted in contrast ratios of 5.7 (tumor/muscle) and 4.8 (tumor/fat pad) for trastuzumab-IRDye800 and 5 (tumor/muscle) and 4.3 (tumor/fat pad) for pertuzumab-IRDye800.

## 4. Discussion

The potential of theranostic agents to influence clinical care is increasingly recognized, especially in the management of cancer. In HER2-positive breast cancer, trastuzumab and pertuzumab are often coadministered because of their synergistic mechanisms of action and routinely given in the neoadjuvant setting in combination with cytotoxic chemotherapy with the goal of achieving complete pathologic response. Yet, this desirable outcome is only realized in approximately 50% of women [19] and emphasizes the importance of detecting residual tumors, including regional lymph nodes. Biopsies are performed to determine HER2 expression in tumors but may not reflect the receptor status due to inter- and intratumor heterogeneity [20, 21]. Moreover, discordance between biopsy-obtained HER2 status in primary and metastatic tumors occurs has significant implications in how patients are managed [22]. Noninvasive imaging of tumors is not subjected to such limitations and has radically changed the landscape of disease staging for cancers such as prostate cancer and [23, 24] neuroendocrine tumors [25, 26] and has shown similar promise in breast cancer. Visualization of HER2-expressing tumors with ^89^Zr-trastuzumab immuno-PET was first reported in 2010 in patients with HER2-positive metastatic breast cancer [27] and has since been used to determine biomarker expression in patients with HER2-negative primary tumors [28, 29], as well as in patients with HER2-positive gastroesophageal cancer [30]. More recently, the first clinical evaluation of ^89^Zr-pertuzumab was performed and showed similar feasibility for HER2-targeted PET imaging in patients with breast cancer [14]. Since the imaging utility of these mAbs is largely based on their established HER2-targeting properties, both agents have the potential to serve as the foundation for fluorescent contrast agents that can guide surgeons in achieving margin-negative resection for often invisible and nonpalpable tumors in the operating room. This could be particularly impactful given the growing integration of NIRF imaging technology in the operating room or endoscopy suites, which has improved the delivery of surgical care and enabled routine use in open, laparoscopic, and robotic surgery. The use of NIRF imaging in breast surgery may be especially appealing due to a low degree of autofluorescence from fat and breast tissue in this spectral range and because of the ease of signal penetration through the relatively low density of fatty tissue.

Despite the roles of both trastuzumab and pertuzumab as frontline agents for treating HER2-positive tumors, preclinical studies have focused almost exclusively on the use of trastuzumab for FGS studies. To the best of our knowledge, the only fluorescent pertuzumab analog that has been tested *in vivo* used the red-shifted dye, Cy5 (ex/em 651/670 nm), which lacks the desirable optical properties of NIRF emitting dyes needed for reliable clinical application [31]. Pertuzumab has been used for NIRF imaging in a dual-labeled format, where it was conjugated to both IRDye800 and ^89^Zr for multimodality imaging of ovarian cancer in mice [32]. However, dual-labeled mAbs are distinct chemical entities in comparison to their fluorescent-only counterparts and may exhibit different physicochemical properties due to the presence of chelating agents used for radiolabeling. In accordance with the development strategy of other FDA-approved mAbs that have been used for FGS in patients [10, 33], this study employed IRDye800 as a single label because of its excellent photochemical properties [34], wide use in clinical studies [7–10, 35–37], and established safety profile [38]. Given the low number of dye molecules per mAb necessary to generate tumor contrast, we anticipated minimal effects on receptor binding efficiency and specificity following conjugation of IRDye800 to the mAbs at D/P ratios of 1-2. This was indeed observed as *in vitro* uptake was highest in BT474 cells, which strongly overexpress HER2, and receptor-mediated as shown by flow cytometry studies with and without blocking. Dual labeling with ^89^Zr permitted further evaluation of cell binding by directly comparing the binding affinities of fluorescent and dual-labeled analogs of each mAb using saturation studies. Findings from those studies showed that dye conjugation had no notable effects on the *K*_d_ values of the ^89^Zr-labeled mAbs. We also measured the percentage of agent uptake in HER2-positive and HER2-negative cell lines and found that, similar to their fluorescent counterparts, uptake of the dual-labeled mAbs correlated with HER2 expression with low nonspecific binding as shown with HER2-negative MCF7 cells. Coincubation with an excess of unlabeled mAb further demonstrated HER2 specificity and indicated suitability of the agents for *in vivo* imaging experiments.

*In vivo* imaging of the fluorescent immunoconjugates showed clear tumor localization in BT474 xenografts with both trastuzumab-IRDye800 and pertuzumab-IRDye800 at 48 h postinjection, while no tumor-specific fluorescence was seen in mice injected with the control mAb. Tumor uptake was notably reduced in MCF7 tumors and is likely a result of the enhanced permeability and retention (EPR) effect. Liver uptake was also present given the role of hepatic clearance as the main elimination route for mAb-based agents. *Ex vivo* imaging was in agreement with the *in vivo* findings, and quantitation of fluorescence values revealed a nearly identical biodistribution profile for trastuzumab-IRDye800 and pertuzumab-IRDye800. Accordingly, no significant differences were seen in the TBRs for the HER2-tageted mAbs. Image contrast in the mammary fat pad is particularly important in order to easily discern lesions from the surrounding fat tissue, and favorable TBR values of 10.1 and 9.3 were obtained for trastuzumab-IRDye800 and pertuzumab-IRDye800 in this site, respectively. In the lungs, a key site for metastatic breast cancer, low background signal resulted in TBRs of 8.7 and 6.9 for trastuzumab-IRDye800 and pertuzumab-IRDye800, respectively. TBRs were >5 for other key organs and demonstrate the potential usefulness of the both immunoconjugates for generating tumor contrast in a surgical setting. Despite differences in NIRF imaging instrumentation and tumor models, these TBRs compare favorably overall to those reported by others with trastuzumab-IRDye800 and further support its use for FGS [39–41]. The results also suggest that pertuzumab may be an effective agent for HER2-targeted FGS based on its similar *in vivo* performance to trastuzumab-IRDye800. Equivalency between the tumor binding and signal intensity of trastuzumab and pertuzumab was previously reported for Cy5-labeled analogs [31]; however, our study is the first to provide such evidence for fluorescent analogs that are IRDye800-labeled and, thus, ideally suited for clinical application. At the microscopic level, histological analysis showed that NIRF readouts from FFPE sections correlated with HER2 positivity as determined by IHC staining. Consistent with *in vitro* and *in vivo* findings, measurement of tissue-associated fluorescence showed identical uptake of trastuzumab-IRDye800 and pertuzumab-IRDye800 in both tumor models, as well as in muscle and fat pad.

The clinical efficacy of trastuzumab and pertuzumab for treating patients with HER2-positive breast cancer has supported the translation of multiple theranostic analogs that build upon their proven targeting properties. This includes the FDA-approved antibody-drug conjugate trastuzumab emtansine (T-DM1) for tumor-targeted chemotherapy delivery and the investigational radiotracers, ^89^Zr-labeled trastuzumab and pertuzumab, for noninvasive imaging. Fluorescent conjugates of trastuzumab and pertuzumab would further extend this theranostic paradigm by providing direct clinical benefit in the operating room. The availability of HER2-targeted FGS agents with different epitope specificities is particularly important as it provides surgeons with the ability to customize FGS protocols according to the type of anti-HER2 therapeutic regimen being administered. The choice of FGS agent could also be complementary, rather than identical, to the treatment regimen in case of target depletion with neoadjuvant systemic treatment. Furthermore, fluorescent immunoconjugates could be used in combination to mimic the therapeutic setting and potentially elicit an additive effect that increases signal intensity in tumor and, thus, enhances detection sensitivity.

## 5. Conclusions

In this study, IRDye800 conjugates of trastuzumab and pertuzumab were evaluated in cells and breast cancer models with varying HER2 expression levels. High receptor specificity was observed *in vitro* and *in vivo* with both agents, along with similar biodistribution profiles. Tumors were readily visualized by NIRF imaging, and high contrast ratios were obtained in surgically relevant tissues, such as the mammary fat pad and lung. These results build on a growing body of clinical evidence that has shown the utility of using FDA-approved mAbs for FGS and may improve clinical management for patients with HER2-positive breast cancer.

## Data Availability

Data can be available upon request.

## Supplementary Materials

Supplementary Materials Supplementary Figure 1: HPLC analysis of trastuzumab-IRDye800 (left) and pertuzumab-IRDye800 (right) performed using 280 nm, 780 nm, and fluorescence channels. Radio-TLC of ^89^Zr-trastuzumab-IRDye800 (left) and ^89^Zr-pertuzumab-IRDye800 (right). Supplementary Figure 2: saturation antigen binding assays. Direct (saturation) radioligand binding to BT474 cells of (a) ^89^Zr-trastuzumab and ^89^Zr-trastuzumab-IRDye800 and (b) ^89^Zr-pertuzumab and ^89^Zr-pertuzumab-IRDye800. Curves were fitted to a 1-site receptor-binding model using Prism Ver. 8.0.2 software. Supplementary Figure 3: competition binding assay of mAb-IRDye800 conjugates to the HER2 antigen on BT474 cells. Cells were incubated with unconjugated or IRDye800-conjugates of trastuzumab and pertuzumab (5 × 10^−6^ to 5 × 10^−2^ mg/mL) and then with ^89^Zr-labeled trastuzumab and pertuzumab. Results show immunoconjugates preserve their antigen specificity and their binding affinity to the HER2 antigen. Supplementary Figure 4: confocal microscopy images of BT474 cells preincubated with unconjugated trastuzumab and pertuzumab and subsequently with the mAb-IR800Dye conjugates. Results show mAb-IRDye800 binding and internalization were significantly reduced in the presence of the blocking doses of the corresponding naked mAbs at 4°C and 37°C, respectively. Supplementary Figure 5: NIRF imaging of trastuzumab-IRDye800 in BT474 xenografts. (a) In vivo images at 48 h p.i. show clear tumor delineation. (b) Ex vivo images of selected tissues confirm selective accumulation in tumors and hepatic clearance. Minimal signal was seen in normal tissues. Supplementary Figure 6: NIRF imaging of pertuzumab-IRDye800 in BT474 xenografts. (a) In vivo images at 48 h p.i. show clear tumor delineation. (b) Ex vivo images of selected tissues confirm selective accumulation in tumors and hepatic clearance. Minimal signal was seen in normal tissues. Supplementary Figure 7: NIRF imaging of IgG-IRDye800 in BT474 xenografts. (a) No tumor-related fluorescence signal was seen 48 h p.i. (b) Ex vivo images showed no notable tumor uptake due to lack of HER2-targeting ability. Minimal signal was seen in normal tissues with the exception of high lung signal in mouse 3. Supplementary Figure 8: NIRF imaging of trastuzumab-IRDye800 in MCF7 xenografts. (a) In vivo images at 48 h p.i. showed negligible uptake of the immunoconjugate in tumors. (b) Ex vivo images of selected tissues confirm in vivo results. Minimal signal was seen in normal tissues. Supplementary Figure 9: NIRF imaging of pertuzumab-IRDye800 in MCF7 xenografts. (a) In vivo images at 48 h p.i. showed negligible uptake of the immunoconjugate in tumors. (b) Ex vivo images of selected tissues confirm in vivo results. Minimal signal was seen in normal tissues. Supplementary Figure 10: NIRF imaging of IgG-IRDye800 in MCF7 xenografts. (a) No tumor-related fluorescence signal was seen 48 h p.i. (b) Ex vivo images showed no notable tumor uptake due to lack of HER2-targeting ability. Minimal signal was seen in normal tissues. Supplementary Figure 11: schematic representation of DFO and IRDye 800 conjugation to trastuzumab. Identical conjugates were prepared with pertuzumab.
