# Supplementary material for: Comparison of HER2-Targeted Antibodies for Fluorescence-Guided Surgery in Breast Cancer
Source: Mol Imaging. 2021 Feb 2;2021:5540569. doi: 10.1155/2021/5540569 (PMC8205604; doi:10.1155/2021/5540569)
Supplement: Supplementary Materials — Supplementary Figure 1: HPLC analysis of trastuzumab-IRDye800 (left) and pertuzumab-IRDye800 (right) performed using 280 nm, 780 nm, and fluorescence channels. Radio-TLC of 89Zr-trastuzumab-IRDye800 (left) and 89Zr-pertuzumab-IRDye800 (right). Supplementary Figure 2: saturation antigen binding assays. Direct (saturation) radioligand binding to BT474 cells of (a) 89Zr-trastuzumab and 89Zr-trastuzumab-IRDye800 and (b) 89Zr-pertuzumab and 89Zr-pertuzumab-IRDye800. Curves were fitted to a 1-site receptor-binding model using Prism Ver. 8.0.2 software. Supplementary Figure 3: competition binding assay of mAb-IRDye800 conjugates to the HER2 antigen on BT474 cells. Cells were incubated with unconjugated or IRDye800-conjugates of trastuzumab and pertuzumab (5 × 10^−6^ to 5 × 10−2 mg/mL) and then with 89Zr-labeled trastuzumab and pertuzumab. Results show immunoconjugates preserve their antigen specificity and their binding affinity to the HER2 antigen. Supplementary Figure 4: confocal microscopy images of BT474 cells preincubated with unconjugated trastuzumab and pertuzumab and subsequently with the mAb-IR800Dye conjugates. Results show mAb-IRDye800 binding and internalization were significantly reduced in the presence of the blocking doses of the corresponding naked mAbs at 4°C and 37°C, respectively. Supplementary Figure 5: NIRF imaging of trastuzumab-IRDye800 in BT474 xenografts. (a) In vivo images at 48 h p.i. show clear tumor delineation. (b) Ex vivo images of selected tissues confirm selective accumulation in tumors and hepatic clearance. Minimal signal was seen in normal tissues. Supplementary Figure 6: NIRF imaging of pertuzumab-IRDye800 in BT474 xenografts. (a) In vivo images at 48 h p.i. show clear tumor delineation. (b) Ex vivo images of selected tissues confirm selective accumulation in tumors and hepatic clearance. Minimal signal was seen in normal tissues. Supplementary Figure 7: NIRF imaging of IgG-IRDye800 in BT474 xenografts. (a) No tumor-related fluorescence s [file 5540569.f1.doc]

**Supplementary Information**

**Comparison of HER2-targeted antibodies for fluorescence-guided surgery in breast cancer**

Solmaz AghaAmiri^1*^, Jo Simien^1*^, Alastair M. Thompson^2,3^, Julie Voss^1^, Sukhen C. Ghosh^1^, Servando Hernandez Vargas^1^, Sarah Kim^1^, Ali Azhdarinia^1§^, and Hop S. Tran Cao^4§^

*Both authors contributed equally to this work.

**Affiliations:**

^1^The Brown Foundation Institute of Molecular Medicine, McGovern Medical School, The University of Texas Health Science Center at Houston, TX 77054, USA.

^2^ Division of Molecular and Clinical Medicine, Ninewells Hospital and School of Medicine, University of Dundee, Dundee DD1 9SY, UK

^3^Michael E. DeBakey Department of Surgery, Baylor College of Medicine, Houston, Texas 77030, USA.

^4^Department of Surgical Oncology, The University of Texas M.D. Anderson Cancer Center, Houston, Texas 77030, USA.

^§^**Corresponding authors:**

Hop S. Tran Cao, MD

The University of Texas M.D. Anderson Cancer Center

Houston, TX 77030

Email: [HSTran@mdanderson.org](mailto:HSTran@mdanderson.org)

Ali Azhdarinia, PhD

The University of Texas Health Science Center at Houston

Houston, TX 77054

Email: [ali.azhdarinia@uth.tmc.edu](mailto:ali.azhdarinia@uth.tmc.edu)


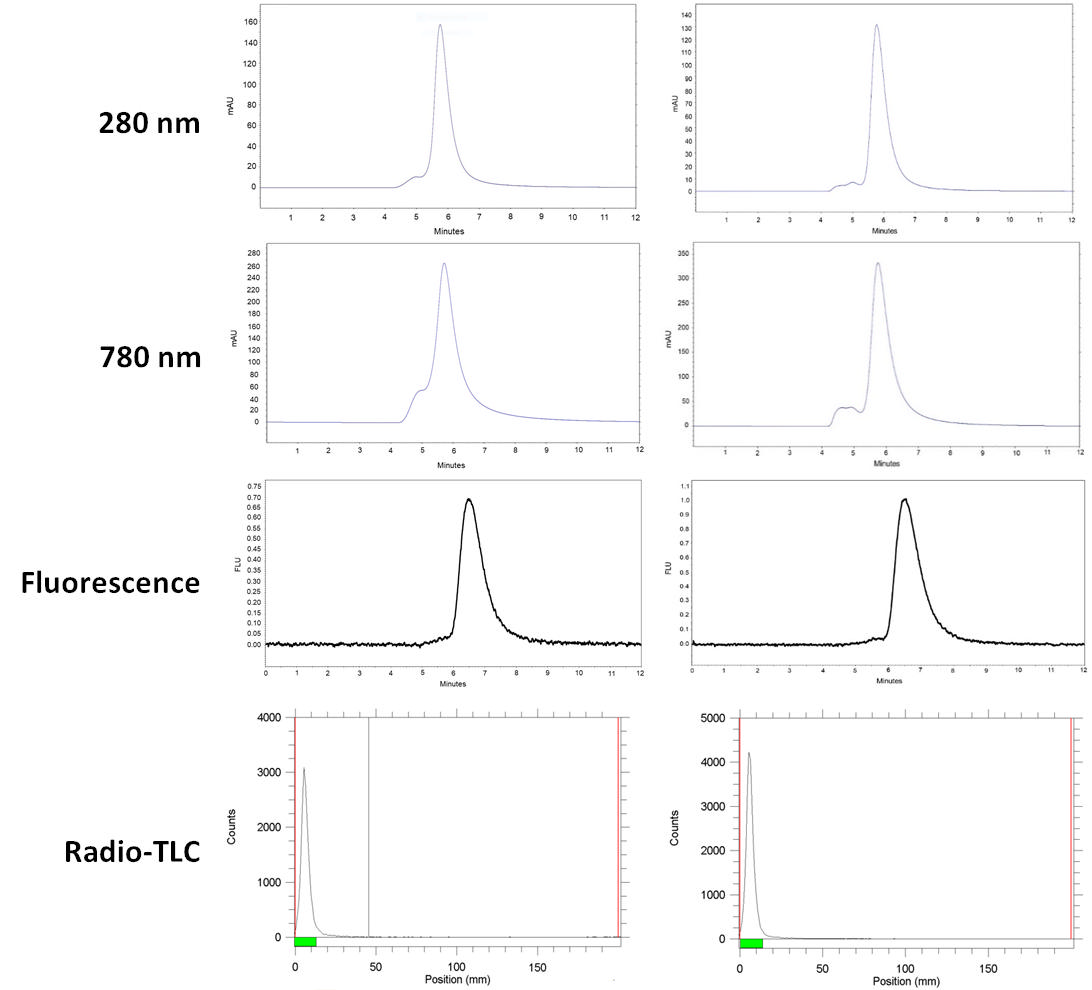


**Supplementary Figure 1.** HPLC analysis of trastuzumab-IRDye800 (left) and pertuzumab-IRDye800 (right) performed using 280 nm, 780 nm, and fluorescence channels. Radio-TLC of ^89^Zr-trastuzumab-IRDye800 (left) and ^89^Zr-pertuzumab-IRDye800 (right).


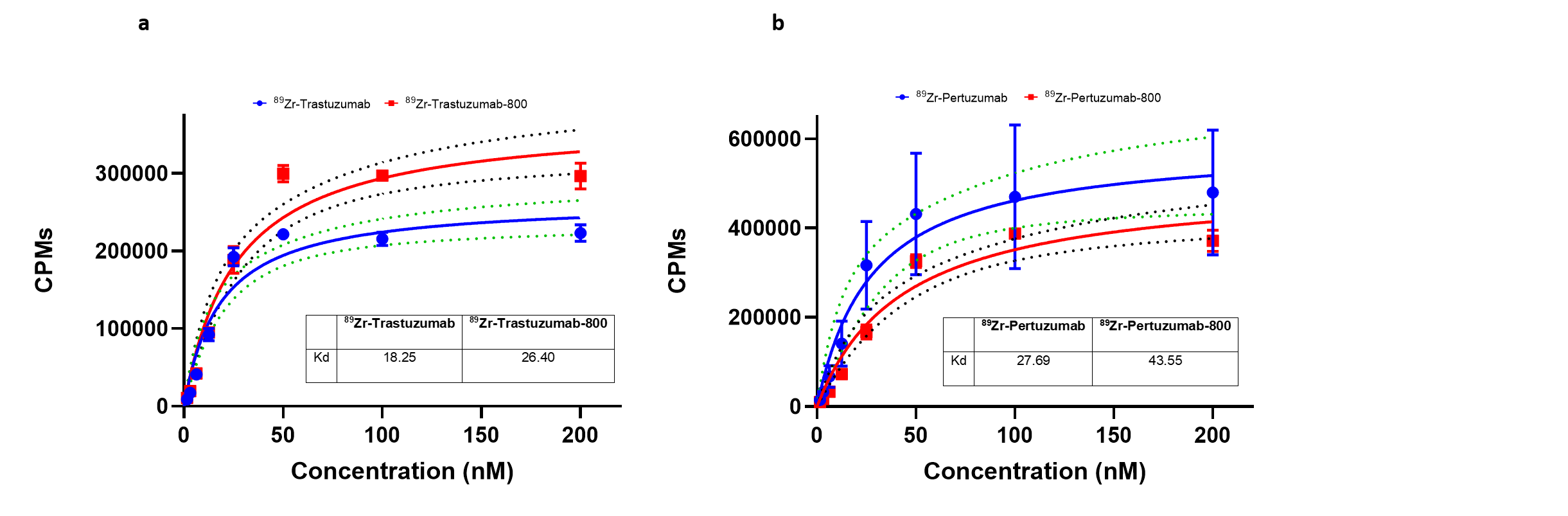


**Supplementary Figure 2.**  Saturation antigen binding assays. Direct (saturation) radioligand binding to BT474 cells of (a) ^89^Zr-trastuzumab and ^89^Zr-trastuzumab-IRDye800, and (b) ^89^Zr-pertuzumab and ^89^Zr-pertuzumab-IRDye800. Curves were fitted to a 1-site receptor-binding model using Prism Ver. 8.0.2 software.

|  | **trastuzumab** | **trastuzumab-800** | **pertuzumab** | **pertuzumab-8000** |
| --- | --- | --- | --- | --- |
| LogIC50 | -4.823 | -4.014 | -4.817 | -4.991 |
| IC50 | 1.503e-005 | 9.692e-005 | 1.524e-005 | 1.022e-005 |

**Supplementary Figure 3.** Competition binding assay of mAb-IRDye800 conjugates to the HER2 antigen on BT474 cells. Cells were incubated with unconjugated or IRDye800-conjugates of trastuzumab and pertuzumab (5 × 10^-6^ to 5 × 10^-2^ mg/mL) and then with ^89^Zr-labeled trastuzumab and pertuzumab. Results shows immunoconjugates preserve their antigen specificity and their binding affinity to the HER2 antigen.


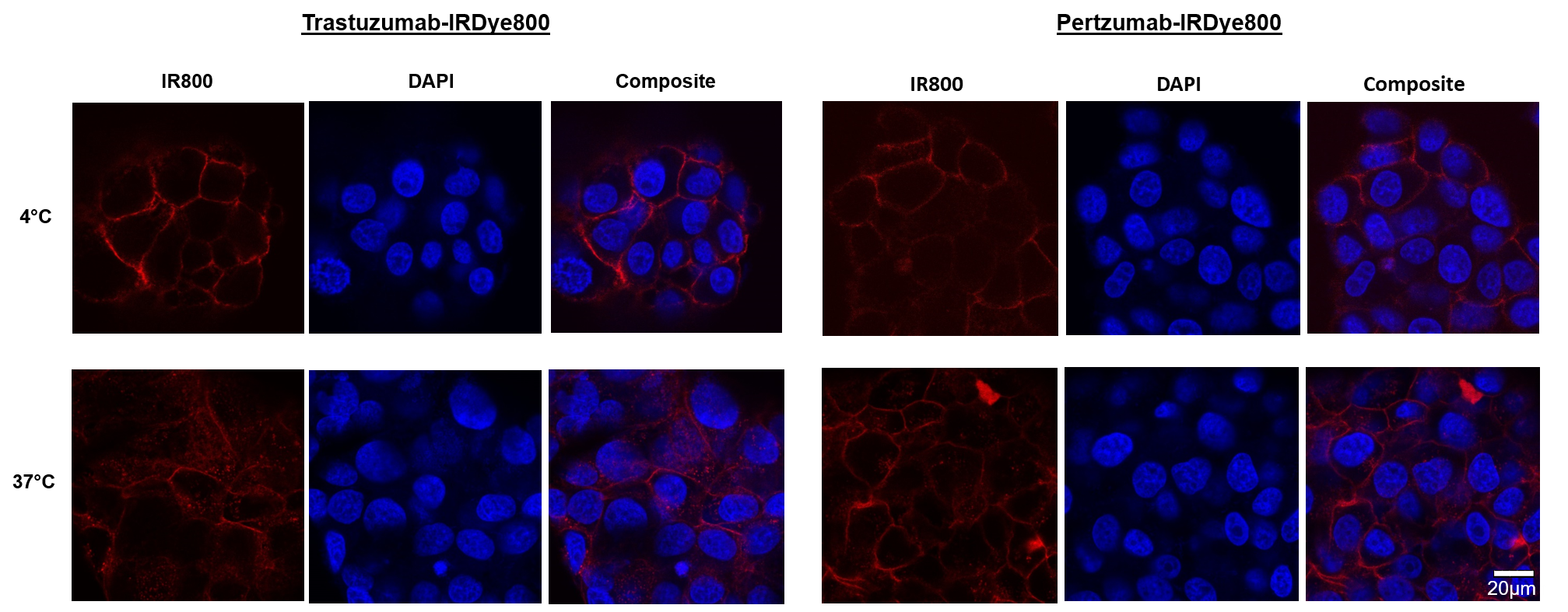


**Supplementary Figure 4.** Confocal microscopy images of BT474 cells preincubated with unconjugated trastuzumab and pertuzumab and subsequently with the mAb-IR800Dye conjugates. Results shows mAb-IRDye800 binding and internalization were significantly reduced in the presence of the blocking doses of the corresponding naked mAbs at 4˚C and 37 ˚C, respectively.


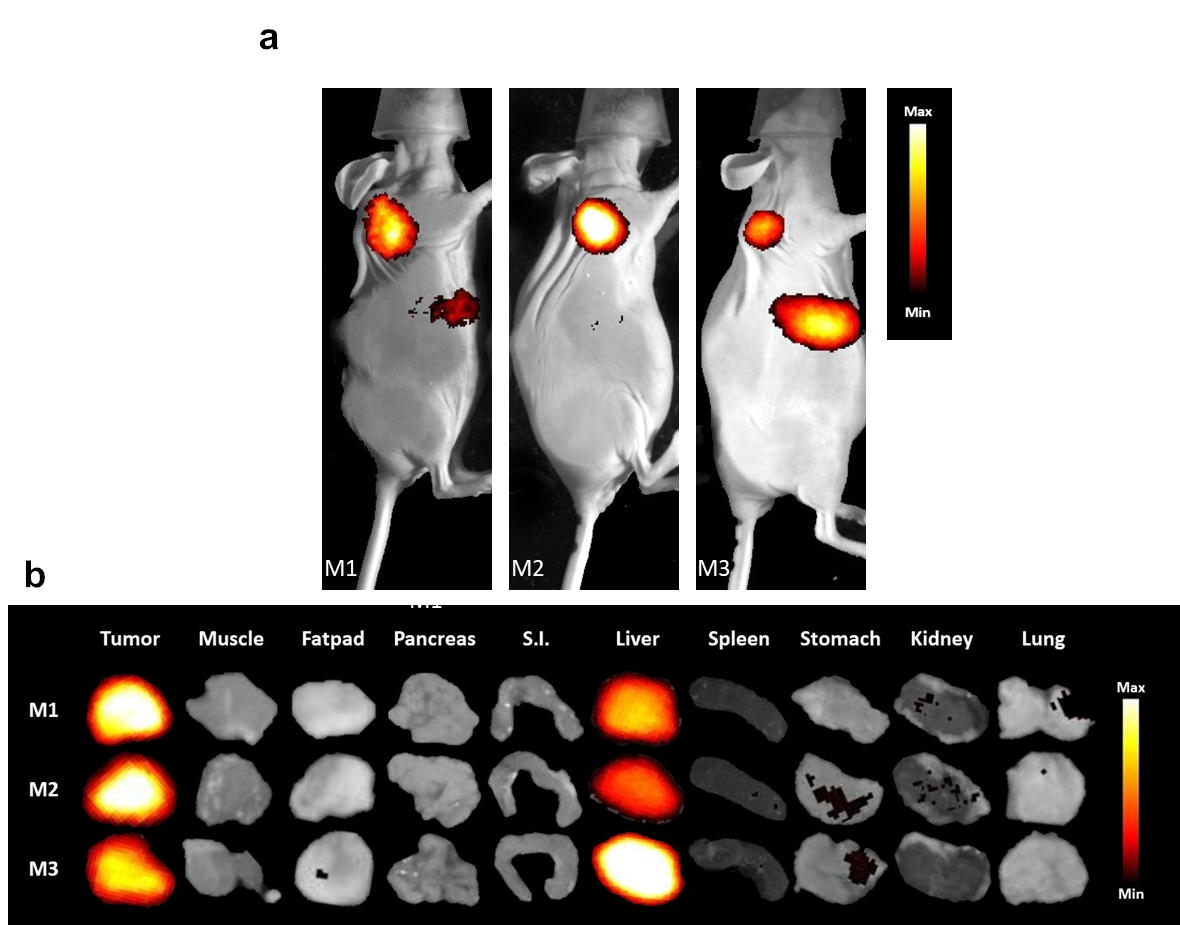


**Supplementary Figure 5.** **NIRF imaging of trastuzumab-IRDye800 in BT474 xenografts.** (a) *In vivo* images at 48 h p.i. show clear tumor delineation. (b) *Ex vivo* images of selected tissues confirm selective accumulation in tumors and hepatic clearance. Minimal signal was seen in normal tissues.

**
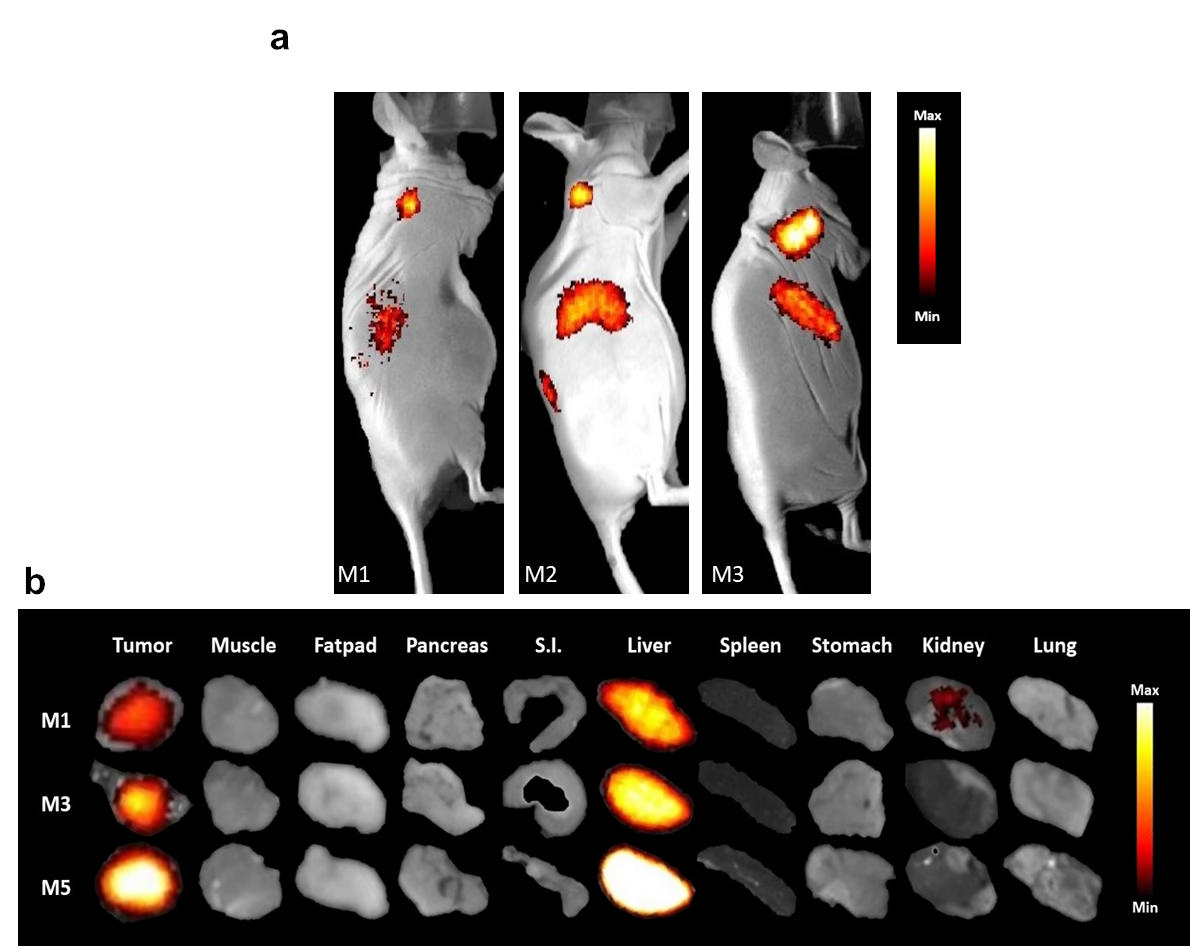
**

**Supplementary Figure 6.** **NIRF imaging of pertuzumab-IRDye800 in BT474 xenografts.** (a) *In vivo* images at 48 h p.i. show clear tumor delineation. (b) *Ex vivo* images of selected tissues confirm selective accumulation in tumors and hepatic clearance. Minimal signal was seen in normal tissues.


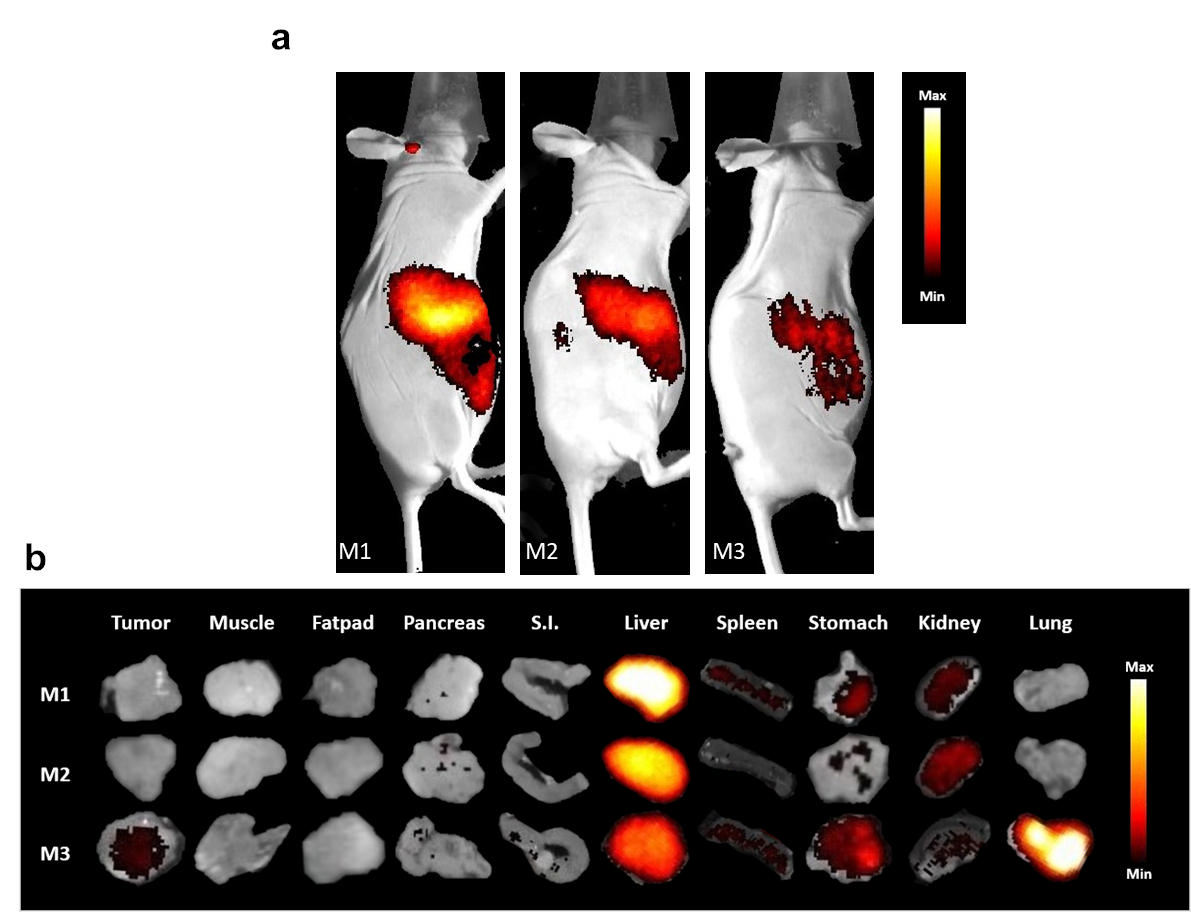


**Supplementary Figure 7. NIRF imaging of IgG-IRDye800 in BT474 xenografts.** (a) No tumor related fluorescence signal was seen 48 h p.i. (b) *Ex vivo* images showed no notable tumor uptake due to lack of HER2-targeting ability. Minimal signal was seen in normal tissues with the exception of high lung signal in mouse 3.

**
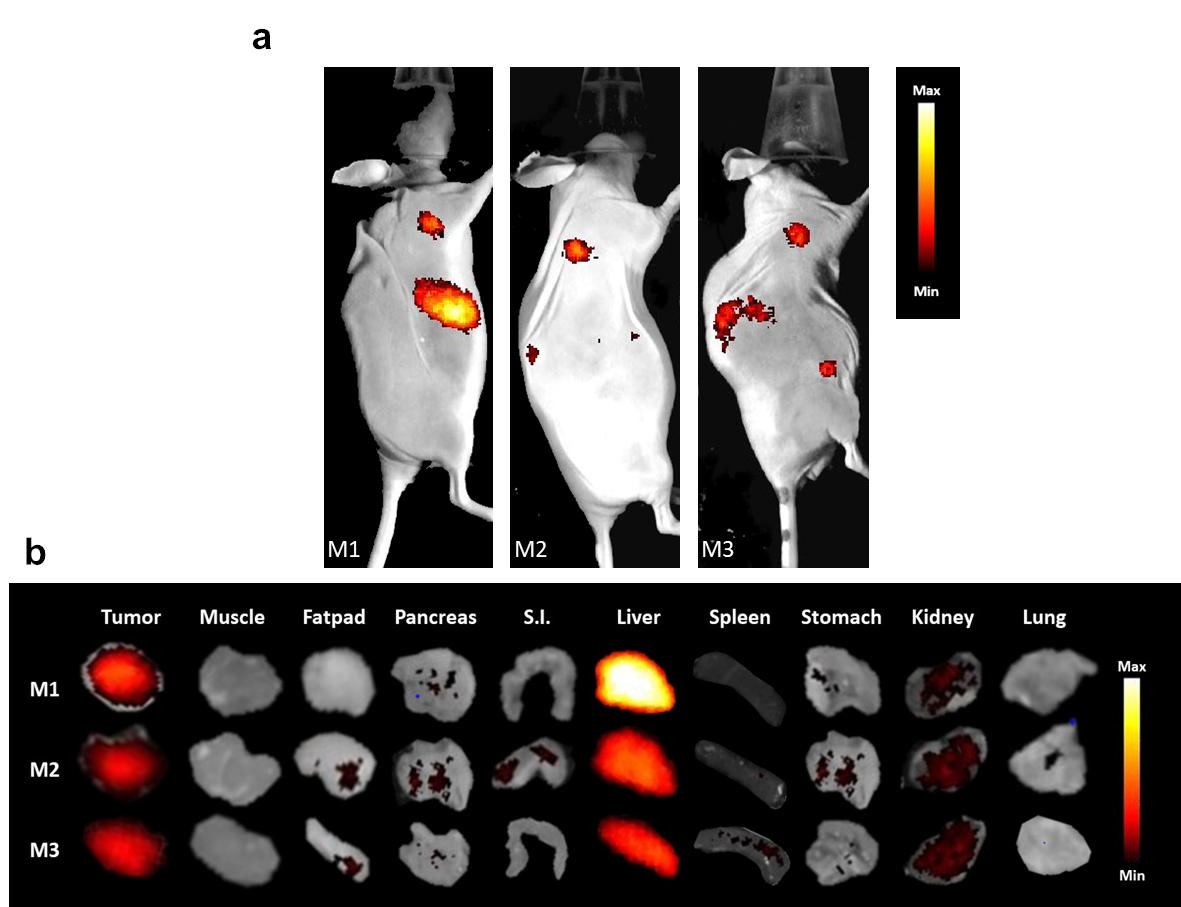
**

**Supplementary Figure 8. NIRF imaging of trastuzumab-IRDye800 in MCF7 xenografts.** (a) *In vivo* images at 48 h p.i. showed negligible uptake of the immunoconjugate in tumors. (b) *Ex vivo* images of selected tissues confirm *in vivo* results. Minimal signal was seen in normal tissues.


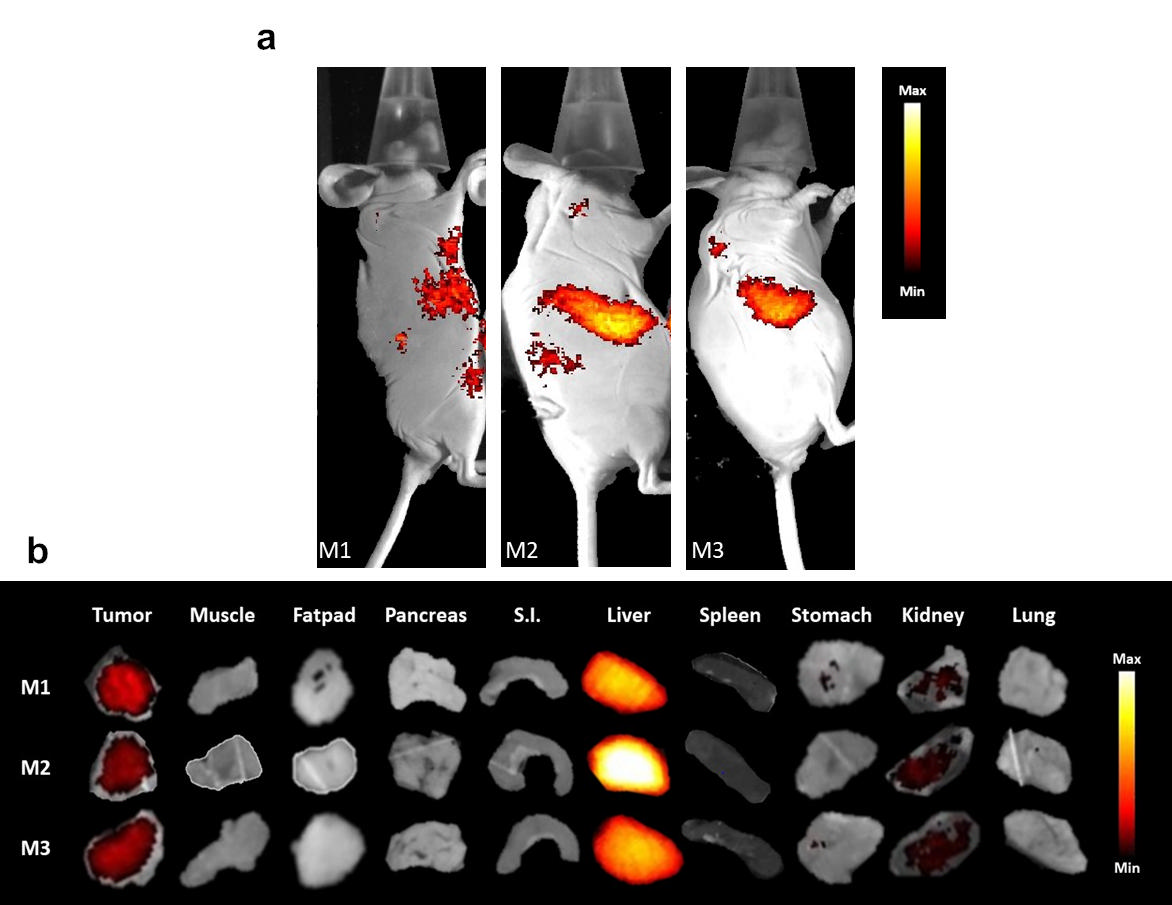


**Supplementary Figure 9. NIRF imaging of pertuzumab-IRDye800 in MCF7 xenografts.** (a) *In vivo* images at 48 h p.i. showed negligible uptake of the immunoconjugate in tumors. (b) *Ex vivo* images of selected tissues confirm *in vivo* results. Minimal signal was seen in normal tissues.

**
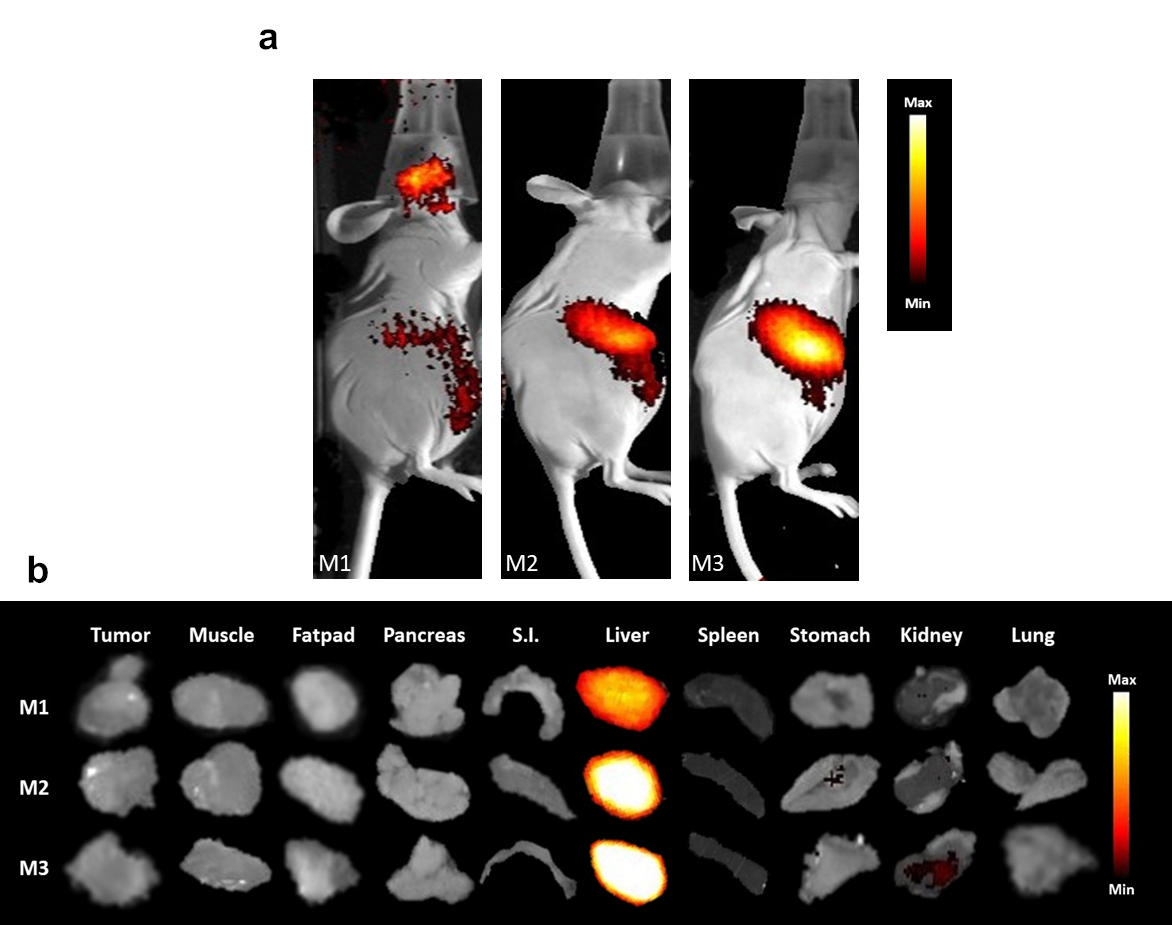
**

**Supplementary Figure 10. NIRF imaging of IgG-IRDye800 in MCF7 xenografts.** (a) No tumor related fluorescence signal was seen 48 h p.i. (b) *Ex vivo* images showed no notable tumor uptake due to lack of HER2-targeting ability. Minimal signal was seen in normal tissues.

**
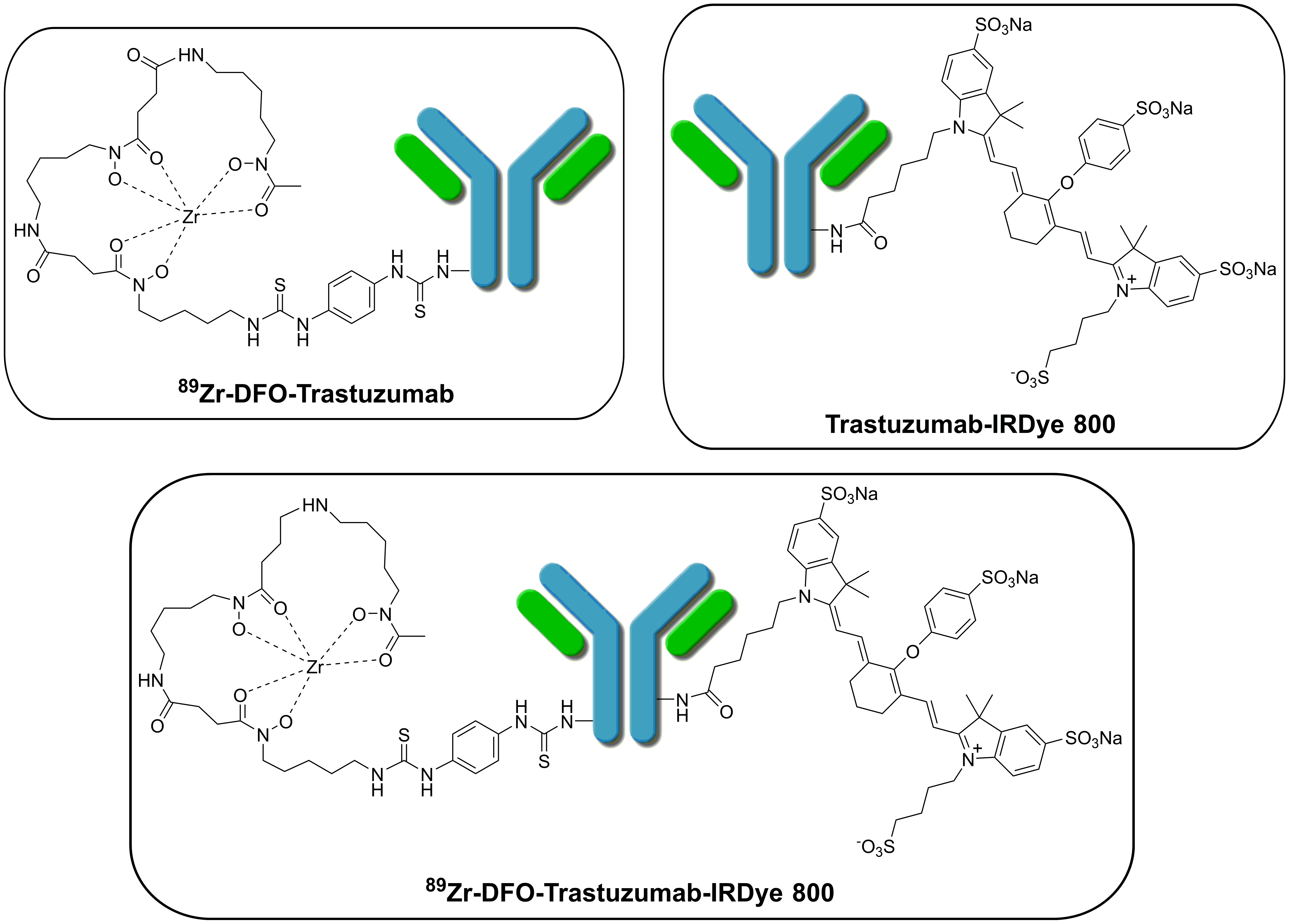
**

**Supplementary Figure 11.** Schematic representation of DFO and IRDye 800 conjugation to trastuzumab. Identical conjugates were prepared with pertuzumab**.**
